# Novel LIPA-Targeted Therapy for Treating Ovarian Cancer

**DOI:** 10.3390/cancers16030500

**Published:** 2024-01-24

**Authors:** Alexia B. Collier, Suryavathi Viswanadhapalli, Rahul Gopalam, Tae-Kyung Lee, Kara Kassees, Karla Parra, Gaurav Sharma, Tanner C. Reese, Xihui Liu, Xue Yang, Behnam Ebrahimi, Uday P. Pratap, Megharani Mahajan, William C. Arnold, Adriana Baker, Chia-Yuan Chen, Scott Terry Elmore, Panneerdoss Subbarayalu, Gangadhara R. Sareddy, Philip T. Valente, Edward R. Kost, Jung-Mo Ahn, Ratna K. Vadlamudi

**Affiliations:** 1 Department of Obstetrics and Gynecology, University of Texas Health San Antonio, San Antonio, TX 78229, USA; alexia.collier@bcm.edu (A.B.C.); viswanadhapa@uthscsa.edu (S.V.); gopalam.rahul@gmail.com (R.G.); yangx1@uthscsa.edu (X.Y.); ebrahimib1@livemail.uthscsa.edu (B.E.); pratap@uthscsa.edu (U.P.P.); mahajanm@uthscsa.edu (M.M.); arnoldw@uthscsa.edu (W.C.A.); bakera5@livemail.uthscsa.edu (A.B.); sareddy@uthscsa.edu (G.R.S.); valentep@uthscsa.edu (P.T.V.); kost@uthscsa.edu (E.R.K.); 2 Mays Cancer Center, University of Texas Health San Antonio, San Antonio, TX 78229, USA; 3 Department of Chemistry and Biochemistry, University of Texas at Dallas, Richardson, TX 75080, USA; leetk92@gmail.com (T.-K.L.); kara.kassees@utdallas.edu (K.K.); chiayuan.chen@utdallas.edu (C.-Y.C.); scott.elmore@utdallas.edu (S.T.E.); jungmo.ahn@utdallas.edu (J.-M.A.); 4 Department of Urology, University of Texas Southwestern Medical Center at Dallas, Dallas, TX 75390, USA; karla.parra@utsouthwestern.edu (K.P.); gaurav.sharma4@utsouthwestern.edu (G.S.); tanner.reese@utsouthwestern.edu (T.C.R.); liuxihui@outlook.com (X.L.); 5 Greehey Children’s Cancer Research Institute, Department of Cell Systems & Anatomy, University of Texas Health San Antonio, San Antonio, TX 78229, USA; subbarayalu@uthscsa.edu; 6 Audie L. Murphy Division, South Texas Veterans Health Care System, San Antonio, TX 78229, USA

**Keywords:** ovarian cancer, lysosomal acid lipase A, LIPA, endoplasmic reticulum stress, ERX-41

## Abstract

**Simple Summary:**

In the United States, ovarian cancer (OCa) is the most lethal of all gynecologic malignancies. Survival rates for OCa with clinically localized disease have been enhanced by presently approved therapies; however, the majority (~90%) of patients with high-grade serous OCa (HGSOC) relapse with incurable metastases. In this investigation, we explored the hypothesis that the elevated basal endoplasmic reticulum stress (ERS) in OCa could be a significant vulnerability, offering a potential strategy to address OCa heterogeneity. We specifically tested the utility of targeting increased ERS in OCa cells by engaging the novel target lysosomal acid lipase A (LIPA) using the unique compound ERX-41. Our findings indicate that ERX-41 is a new targeted therapy that specifically targets the LIPA protein through a distinct mechanism of action. Furthermore, our results reveal that the binding of ERX-41 to LIPA leads to the induction of ERS and cell death in OCa cells.

**Abstract:**

Ovarian cancer (OCa) is the most lethal form of gynecologic cancer, and the tumor heterogeneities at the molecular, cellular, and tissue levels fuel tumor resistance to standard therapies and pose a substantial clinical challenge. Here, we tested the hypothesis that the heightened basal endoplasmic reticulum stress (ERS) observed in OCa represents an exploitable vulnerability and may overcome tumor heterogeneity. Our recent studies identified LIPA as a novel target to induce ERS in cancer cells using the small molecule ERX-41. However, the role of LIPA and theutility of ERX-41 to treat OCa remain unknown. Expression analysis using the TNMplot web tool, TCGA data sets, and immunohistochemistry analysis using a tumor tissue array showed that LIPA is highly expressed in OCa tissues, compared to normal tissues. ERX-41 treatment significantly reduced the cell viability and colony formation ability and promoted the apoptosis of OCa cells. Mechanistic studies revealed a robust and consistent induction of ERS markers, including CHOP, elF2α, PERK, and ATF4, upon ERX-41 treatment. In xenograft and PDX studies, ERX-41 treatment resulted in a significant reduction in tumor growth. Collectively, our results suggest that ERX-41 is a novel therapeutic agent that targets the LIPA with a unique mechanism of ERS induction, which could be exploited to treat heterogeneity in OCa.

## 1. Introduction

Ovarian cancer (OCa) is the deadliest of all gynecologic cancers in the United States [1,2]. Currently approved therapies (such as cytoreductive surgery, a combination of platinum-based chemotherapies, and PARP inhibitors) have improved OCa survival for clinically localized disease and are widely used [3,4]. Despite these advances, the majority (~90%) of patients with high-grade serous OCa (HGSOC) relapse with incurable metastases. Unfortunately, the overall survival for metastatic OCa is dismal (<20% 5-year survival) and has not changed significantly for several decades [5]. Intratumoral and intertumoral heterogeneities of OCa at the molecular, cellular, and tissue levels drive tumor resistance to standard therapies and represent a significant clinical challenge [6]. There is thus a desperate and unmet need for new therapeutic approaches to target fundamental and critical pathways in OCa that will effectively cause OCa cell death and improve the survival of OCa patients.

OCa cells have a high growth rate and have a sustained and enhanced demand for de novo protein synthesis, folding, and maturation [7]. Since proper folding and post-translational maturation of most cellular proteins occur in the endoplasmic reticulum (ER) [8], OCa cells have a high basal level of ER stress (ERS). The hostile environmental conditions, such as hypoxia, oxidative stress, and early exposure to chemotherapy regimens, jeopardize the fidelity of protein folding in the ER and further enhance ERS [9]. Several genome-scale genetic screens with short hairpin RNAs (shRNAs) identified components of ERS and unfolded protein response (UPR) pathways as targets of vulnerability in OCa [10,11,12]. While the activation of UPR enables OCa cells to viably compensate for higher basal levels of ERS, uncompensated ERS can be lethal to cells through CCAAT-enhancer binding protein homologous protein (CHOP) pathway induction and the ensuing apoptosis [13]. Despite published evidence supporting ERS as a vulnerability in OCa, the lack of a clinically translatable small molecule that exploits ERS vulnerability in OCa represents a major gap.

Recently, we identified a small molecule, ERX-41, that dramatically enhances ERS in cancer cells, leading to apoptotic cell death [14]. Importantly, ERX-41 is not toxic to normal cells, which have a low basal ERS, or to OCa cells, with the pharmacologic attenuation of their basal ERS levels. Using CRISPR knockout (KO) screens, we identified the lysosomal acid lipase A gene (LIPA) as the target of ERX-41 [14]. The knockdown of LIPA expression abrogates the ability of ERX-41 to induce ERS and apoptosis, while the reconstitution of LIPA expression restores responsiveness to ERX-41 [14].

In this study, we evaluated the efficacy of LIPA as a molecular target and explored the potential of ERX-41 as a novel treatment for OCa using both primary and established OCa models. Through the analysis of public databases and tumor tissue arrays, we found an elevated expression of LIPA in OCa tissues. Using 15 distinct OCa model cells, patient-derived organoids, and xenograft models, we demonstrated the capacity of ERX-41 to effectively reduce the growths of OCa model cells and tumors. Using mechanistic and biochemical assays, we validated that ERX-41 induces ERS in OCa cells in a LIPA-dependent manner. Overall, our findings collectively suggest the potential utility of ERX-41 as an innovative therapeutic approach for the treatment of OCa.

## 2. Materials and Methods

### 2.1. Cell Culture and Reagents

The OVCAR3 (HGSOC), ES2, OV56 (LGSOC), SKOV3 (CCOC), A2780 (EOC), and OV90 (MOC) cell lines were obtained from the American Type Culture Collection (ATCC, Manassas, VA, USA) and cultured using the media suggested by ATCC. The OVSAHO (HGSOC) cell line was acquired from AcceGen™ and cultured in RPMI-1640 media supplemented with 10% FBS (Sigma, St. Louis, MO, USA) and Gibco™ Antibiotic-Antimycotic. OV56 (LGSOC) and OVCAR4 (HGSOC) cells were purchased from Sigma. OVCAR8 (LGSOC) cells were obtained from the NCI DCTD repository. The classification of existing cells into subtypes was based on recent publications [15,16]. Primary OCa cells derived from the tumor (OCa30, OCa39, and OCa49), as well as ascites (Asc25, Asc28, Asc34, and Asc39), were obtained from the Ob/Gyn tissue core that received IRB approval (Supplementary Table S1). All the model cells employed were free of mycoplasma. The cells’ identities were additionally confirmed by STR DNA fingerprinting. The antibodies for p-eIF2α, eIF2α, PERK, CHOP, and ATF4 were acquired from Cell Signaling Technology (Danvers, MA, USA). The Ki67 antibody (ab1667) was acquired from Abcam (Cambridge, MA, USA). The LAL (LIPA) antibody (sc-58374) was obtained from Santa Cruz (Dallas, TX, USA). LIPA-KO SKOV3 cells were created using the process of lentiviral transduction, utilizing a validated gRNA that targets the LIPA gene, as described [14]. ERX-41 was synthesized using the protocol described in our earlier publication [14].

### 2.2. Cell Viability, Colony Formation, Cell Invasion, and Apoptosis Assays

The cell viability rates of the control and ERX-41-treated cell lines were evaluated using MTT assays, following the earlier established protocol [17,18]. The impacts of ERX-41 on colony formation and apoptosis were assessed using established methodologies, as previously described [19]. The effect of ERX-41 on cell invasion was determined by using the Corning BioCoat Growth Factor Reduced Matrigel Invasion Chamber assay. Briefly, SKOV3 and Asc25 cells were treated with vehicle or ERX-41 (1 µM) for 22 h, and invaded cells were determined according to the manufacturer’s protocols.

### 2.3. Western Blotting and Quantitative Real Time-PCR

The OCa cells were lysed using RIPA lysis buffer that contained inhibitors for proteases and phosphatases. Subsequently, we performed Western blotting analysis. The activation of known ERS genes was validated using quantitative PCR (RT-qPCR), with total RNA isolated from OCa39, OCa30, and OVCAR3 cells using the Qiagen RNA extraction kit (Valencia, CA, USA). The primer sequences are detailed in Supplementary Table S2. The RT-qPCR analysis was conducted utilizing the CFX96 real-time PCR system from Bio-Rad (Hercules, CA, USA), with the utilization of the SYBR Green Master Mix provided by Applied Biosystems™ (Waltham, MA, USA).

### 2.4. RT-PCR for XBP1 mRNA Splicing Assay

OCa cells were treated with ERX-41 (1 µM) for 0, 2, 3, 4, and 6 h, and total RNA was extracted using the RNeasy mini kit using the manufacturer’s protocol. First-strand cDNA was synthesized using the SuperScript III First-Strand kit (Invitrogen, Waltham, MA, USA). To evaluate relative expression levels of unspliced and spliced XBP1, semi-quantitative RT-PCR analysis was performed using PCR SuperMix (Invitrogen). XBP1u/XBP1s cDNA fragments were amplified by PCR using the following primers: XBP1u/XBP1s-F-5′CCTGGTTGCTGAAGAGGAGG3′ and XBP1u/XBP1s-R-5′CCATGGGGAGATGTTCTGGAG3′. GAPDH was used as a loading control, with primers as follows: 5′-GGGTCAGAAGGATTCCTATG-3′ and 5′-GGTCTCAAACATGATCTGGG-3′. PCR products were analyzed on a 3% agarose gel.

### 2.5. Transmission Electron Microscopy (TEM) Studies

OCa cells treated with control and ERX-41 (1 µM for 8 h) were detached using trypsin and then rinsed with PBS. The cells were subsequently fixed using a solution of 4% formaldehyde and 1% glutaraldehyde in PBS. The cells were subsequently treated with a 2% solution of OsO4, dehydrated using ethanol, and then implanted in Poly/Bed^®^ 812 (Polysciences, Warrington, PA, USA). The ultrathin slices were prepared using a Leica ultramicrotome (Wetzlar, Germany) and subsequently stained with uranyl acetate and lead citrate. The imaging process was carried out using a JEOL JEM-1400 transmission electron microscope (Tokyo, Japan).

### 2.6. Tissue Microarray (TMA) and Immunohistochemistry (IHC)

Ovarian tissue microarray (OV1021) was purchased from TissueArray (Derwood, MD, USA). Ovary tissue microarray with normal tissue contained 65 cases of cystadenocarcinoma (21 serous and 44 mucinous), 22 endometrioid adenocarcinomas, 3 clear cell carcinomas, and 5 normal/benign tissues, with a single core per case. IHC analysis was performed as previously described [14]. TMA was probed with the LIPA antibody. IHC on xenograft tissues using the Ki67 antibody was performed as described previously [14]. The proliferative index was calculated using Ki67-positive cells in five randomly selected microscopic fields at 20× per slide.

### 2.7. Xenograft Studies

Cell-based xenografts were initially generated using a subcutaneous injection of 10^6^ SKOV3 or OVCAR8 cells, as described [20]. The female SCID mice were then subcutaneously implanted with tumor tissue measuring 2 mm^3^. Once tumors reached a size of ~150 mm^3^, the mice were divided into control and treatment groups, with each group having *n* = 6 tumors. The mice were treated with vehicle (0.3% hydroxypropyl cellulose) or ERX-41 in vehicle at a dosage of 10 mg/kg intraperitoneally for 5 days per week. Tumor volume was measured at regular intervals of 3–4 days using a digital caliper. At the end of the experiment, the tumors were extracted and processed for IHC analyses.

### 2.8. Patient-Derived Organoid Studies

Patient-derived organoids (PDOs) were generated from de-identified OCa tissues obtained from the UTHSA Ob/Gyn tissue core. Organoids were created utilizing a procedure that was previously published [21]. The organoid cultures were exposed to a series of dilutions of either DMSO (vehicle) or ERX-41 at different concentrations in triplicate. Cell viability was assessed after a 7-day treatment period using the Promega^®^ CellTiter-Glo^®^ 3D-Superior Cell Viability Assay reagent (Promega, Madison, WI, USA). The measurement of luminescence intensity was conducted using a GloMax^®^ Discover System (Promega, Madison, WI, USA).

### 2.9. Databases and Statistical Analyses

To analyze changes in LIPA levels in OCa, we used the TNM plot analysis tool (https://tnmplot.com/analysis/, accessed on 5 September 2023), which compares gene expression variations between tumor and normal tissues using a validated database [22]. Progression-free survival analysis of OCa patients was performed using the Kaplan–Meier plotter [23]. Utilizing GraphPad Prism 9 software, statistical differences between groups were examined using unpaired Student’s *t*-test and one-way ANOVA. All data depicted in plots are denoted as means ± standard error (SE). A *p*-value less than 0.05 was deemed to indicate statistical significance.

## 3. Results

### 3.1. LIPA Expression Upregulated in OCa and Correlated with Poor Survival

**Figure 1:**
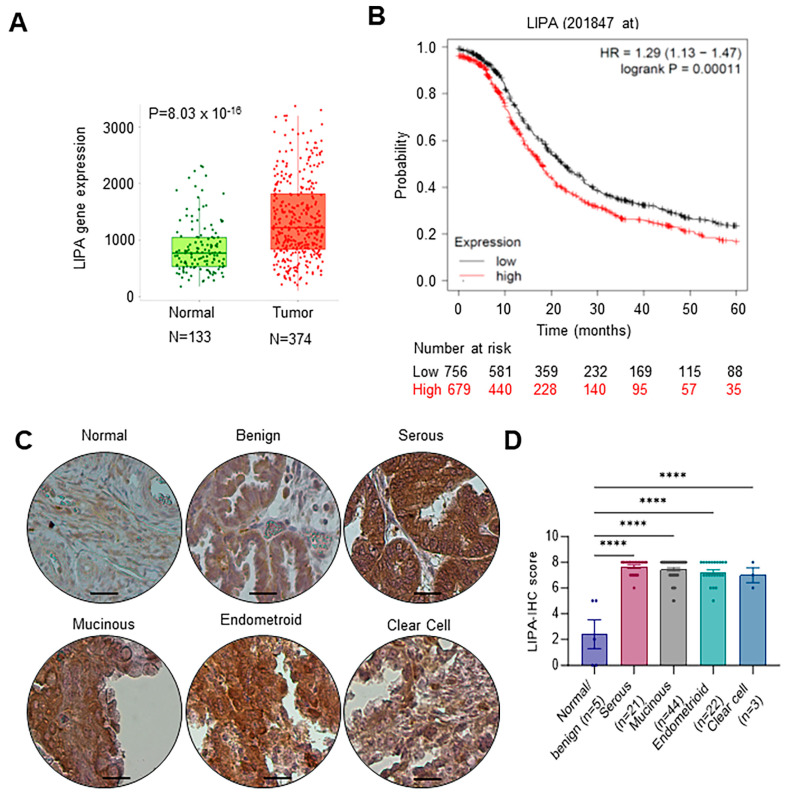
LIPA is overexpressed in OCa. (**A**) Boxplots of LIPA gene expression in normal (*n* = 133) and tumor (*n* = 374) gene array data. Data was obtained from the TNMplot database. (**B**) Association of LIPA gene expression with the overall survival of OCa patients using KM plotter. (**C**,**D**) Samples from 4 subtypes of OCa and normal ovarian tissue were evaluated for LIPA expression using tissue microarray, with representative images (**C**) and quantitation (**D**). Scale bar represents 100 µm. Data are represented as mean ± SEM. **** *p* < 0.0001.

We utilized the TNM plot analysis tool, which allows for the comparison of gene expression variations between tumor and normal tissues using a validated database [22], to investigate changes in LIPA levels in OCa. The findings indicate that LIPA has a significantly elevated expression level in ovarian tumors when compared to normal tissues (Figure 1A). Analysis of TCGA data using the Kaplan–Meier plotter showed a negative correlation between LIPA expression and progression-free survival in patients with OCa (Figure 1B). We further validated the increased expression of LIPA in OCa using IHC with the LIPA antibody by employing a tissue microarray (TMA) consisting of 21 serous, 44 mucinous, 22 endometrioid adenocarcinoma, 3 clear cell carcinoma tumors and 5 normal/benign ovarian tissues. The results indicated that the expression of LIPA is increased in all subtypes of ovarian tumors in comparison to normal/benign ovarian tissue (Figure 1C,D).

### 3.2. ERX-41 Reduced Cell Viability, Colony Formation, and Invasion and Induced Apoptosis of OCa Cells

**Figure 2:**
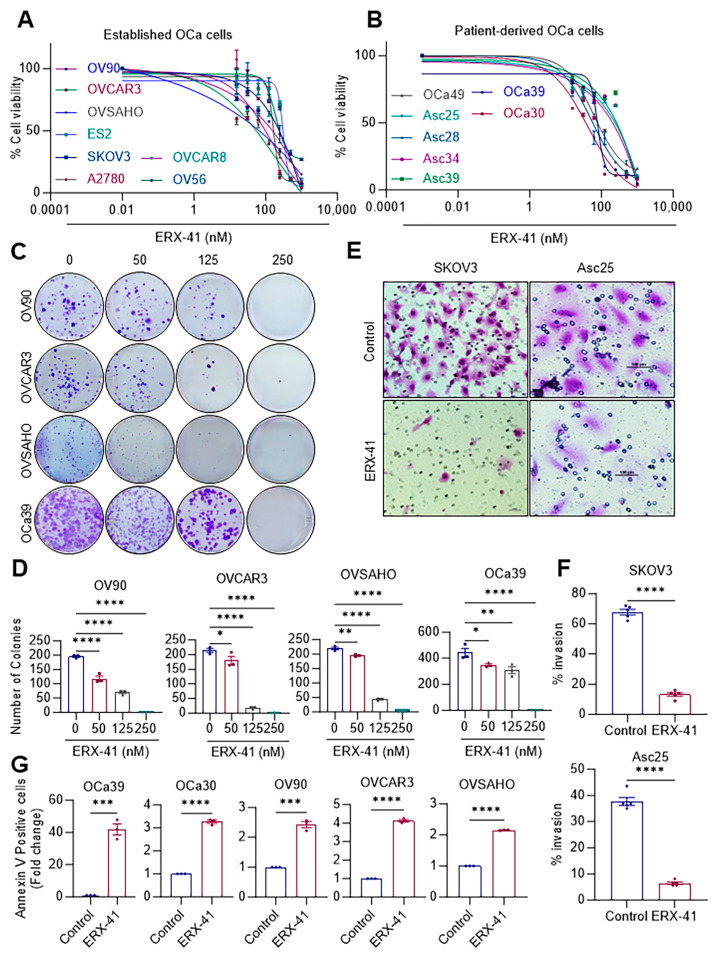
ERX-41 is potent against OCa cells in vitro. (**A**,**B**) Effect of ERX-41 on the cell viability of established (**A**) and patient-derived primary OCa cells (**B**) was determined using MTT assay. (**C**,**D**) Established and primary OCa cells were treated with the indicated doses of ERX-41, and the colony formation was examined after 14 days (**C**). The numbers of colonies were quantified and were shown as bar graphs in the lower panel (**D**). (**E**,**F**) The effect of ERX-41 on the cell invasion of OCa cells was determined using BioCoat Matrigel invasion chamber assays. Images are shown in panel (**E**), scale bar represents 100 µm (**E**), and the quantitation of the percentage of cells invaded is shown in panel (**F**). (**G**) The effects of ERX-41 (500 nmol/L) on the apoptosis of established and patient-derived OCa cells were determined using Annexin V/PI staining. Data are represented as mean ± SEM. * *p* < 0.05; ** *p* < 0.01; *** *p* < 0.001; **** *p* < 0.0001.

Our recent studies have found a small chemical molecule, ERX-41, that binds to LIPA, interferes with its functions in ER, and promotes ERS [14]. In order to investigate the ability of ERX-41 to overcome the commonly observed tumor heterogeneity in OCa, we used the MTT-based cell viability assay. More precisely, we employed 15 OCa cell lines that encompass all four distinct subtypes of OCa, comprising eight established, three primary, and four ascites-derived OCa cell lines (Figure 2A,B). The treatment of ERX-41 resulted in a significant decrease in the cell viability of 15 OCa cell lines, with an inhibitory concentration (IC_50_) ranging from 100 to 200 nM (Figure 2A,B). We used Western blot analysis to determine the LIPA expressions in three OCa cells with high expression and three OCa cells with low expression in order to investigate whether there was a correlation between the levels of LIPA expression and the cellular activity of ERX-41. The expression of LIPA and the response to ERX-41 therapy was correlated in five out of six OCa cells, with the exception of the OVSAHO cell line (Supplementary Figure S1). These results suggest that in addition to LIPA, other cellular factors, such as levels of ERS in the cells, may also contribute to the activity of ERX-41. Further, ERX-41 treatment significantly decreased the long-term colony formation ability (Figure 2C,D) and invasion of OCa cells (Figure 2E,F). In addition, ERX-41 greatly increased the apoptosis of OCa cells, as seen in Figure 2G. Overall, these findings indicate that ERX-41 decreases the cell viability, survival, and invasion of OCa cells and enhances apoptosis in OCa.

### 3.3. ERX-41 Treatment Promoted Activation of ER Stress Pathways

**Figure 3:**
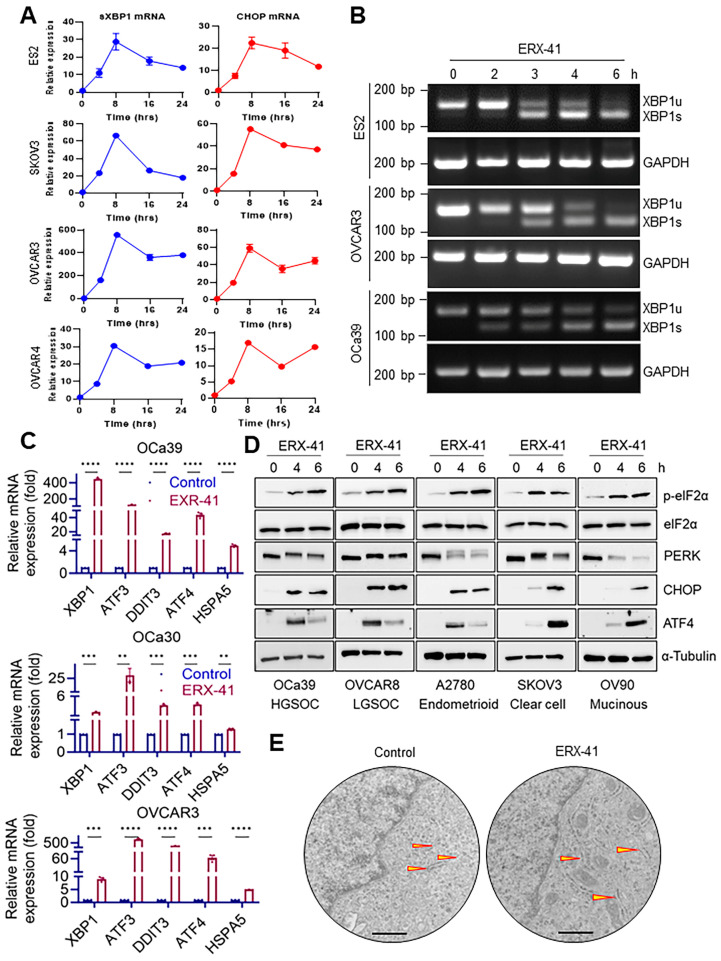
ERX-41 induces ER stress (ERS) in OCa. (**A**) The time course of the effects of ERX-41 (1 µM) on the mRNA expressions of ERS genes, XBP1s, and CHOP in ES2, SKOV3, OVCAR3, and OVCAR4 cells. (**B**) RT-PCR analysis shows the time course of the effects of ERX-41 (1 µM) on the expression of XBP1 (unspliced (XBP1u) and spliced (XBP1s)) in ES2, OVCAR3, and OCa39 cells. (**C**) OCa cells were treated with ERX-41 (1 µM, 6 h), and the statuses of ERS genes were measured by RT-qPCR. (**D**) OCa cells were treated with ERX-41 for indicated time points, and the statuses of the activations of UPR components were analyzed by Western blotting. (**E**) Transmission electron microscopy of OVCAR8 cells shows the effects of vehicle and ERX-41 treatments on subcellular structures at 8 h; ER is outlined with yellow arrowheads. Scale bar represents 400 nm. Data are represented as mean ± SEM. ** *p* < 0.01; *** *p* < 0.001; **** *p* < 0.0001.

Our previous research showed that ERX-41 is a powerful stimulator of ERS [14]. We investigated the potential of ERX-41 to induce ERS in the context of OCa heterogeneity. This was performed by employing various OCa models and assessing the activation of markers that reflect the activation of ERS pathways. At first, we performed a time course study utilizing RT-qPCR. The findings demonstrated a strong and consistent increase in ERS markers (>15-fold CHOP and >10-fold XBP1s) in four different OCa cells caused by ERX-41 during a timeframe of 8–16 h (Figure 3A). We subsequently confirmed the activation of XBP1s using an RT-PCR-based splicing assay. Results demonstrated that the XBP1 transcript underwent splicing within 3 h of being treated with ERX-41 (Figure 3B; Supplementary Figure S2A). We further confirmed the activation ERS genes by performing RT-qPCR in three different OCa cells, and the results demonstrated that treatment with ERX-41 increased the levels of ERS markers (Figure 3C). In addition, treatment with ERX-41 induced the activation of downstream targets associated with ERS pathways, such as PERK, p-eIF2α, CHOP, and ATF4, in several OCa models (Figure 3D; Supplementary Figure S2B–D). We used transmission electron microscopy to assess the morphological alterations in the ER in OCa cells after ERX-41 treatment. The results demonstrated that treatment with ERX-41 caused significant changes in the structure of the ER lumen, specifically considerable enlargement of the ER, which is in line with its ability to induce ERS, as shown in Figure 3E. Collectively, these results demonstrate that ERX-41 has a strong potential to trigger ERS in OCa cell lines that have different genetic backgrounds, confirming that ERX-41 exploits a shared weakness in OCa.

### 3.4. ERX-41-Mediated Effects Require LIPA in OCa Cells

**Figure 4:**
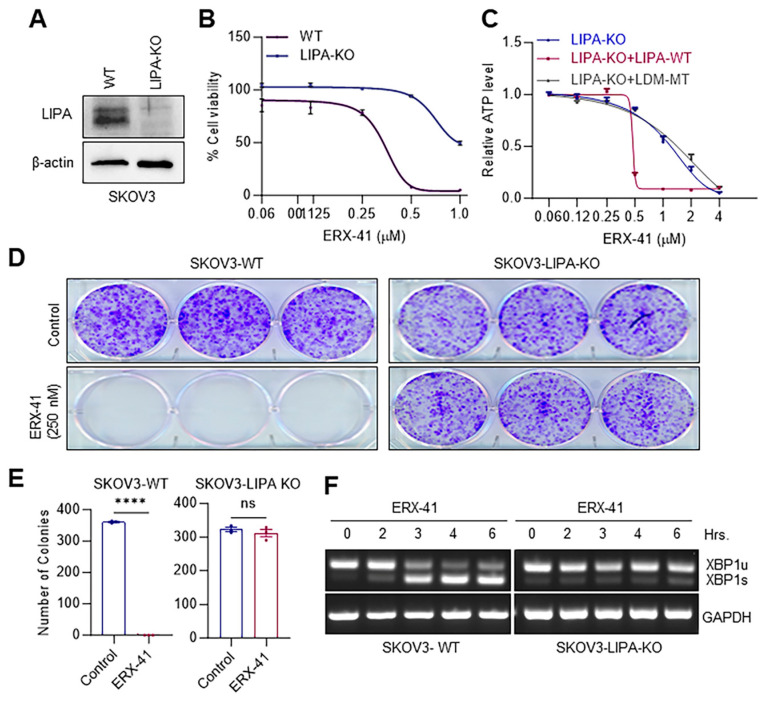
LIPA is the target of ERX-41. (**A**) LIPA-KO in SKOV3 cells was confirmed using Western blotting. (**B**) The effect of the KO of LIPA in SKOV3 cells on the dose–response curve to ERX-41 was determined using CellTiter-Glo assays. (**C**) Dose–response curves to increasing concentrations of ERX-41 in KO, KO + WT, and KO + MT SKOV3 cells were performed by the CellTiter-Glo assay. (**D**) The effect of LIPA-KO on the activity of ERX-41 in reducing the colony formation was determined. (**E**) Quantitation of colonies are shown. (**F**) SKOV3-WT and LIPA-KO cells were treated with ERX-41 for indicated time points, and the status of the splicing of XBP1 was measured by RT-PCR. Data are represented as mean ± SEM. **** *p* < 0.0001; ns, not significant.

To confirm the specificity of the target, we utilized CRISPR-Cas9 knockout (KO) to create a cell line (SKOV3) with a LIPA-KO (Figure 4A; Supplementary Figure S3A). The LIPA-KO in SKOV3 cells reduced the sensitivity of these cells to ERX-41 (Figure 4B). To further confirm the specificity of LIPA for ERX-41 activity, we restored LIPA expression in LIPA-KO SKOV3 cells by transfecting them with LIPA WT or LIPA LDM mutant (LXXLL domain MT that lacks an ERX-41 binding site) expression plasmids [14]. The restoration of LIPA WT but not MT in LIPA-KO cells enabled ERX-41 to effectively reduce cell viability, as seen in Figure 4C. We also confirmed the necessity of LIPA in ERX-41-mediated reduction in colony formation and the generation of ERS, as assessed by the induction of XBP1 transcripts. The results showed that LIPA is essential for reducing the formation of colonies by ERX-41 (Figure 4D,E) and for the induction of XBP1s by ERX-41 (Figure 4F; Supplementary Figure S3B). Collectively, these findings validate the essential role of LIPA in mediating the effects of ERX-41 in OCa cells.

### 3.5. ERX-41 Reduced Cell Viability of Patient-Derived OCa Organoids

**Figure 5:**
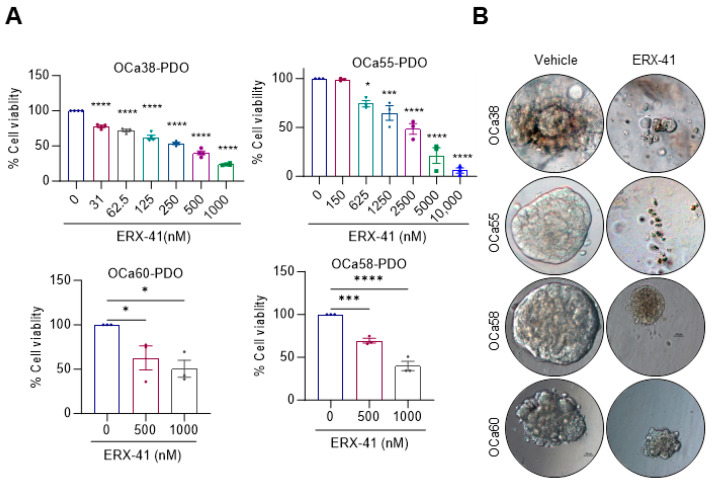
ERX-41 effectively reduces the growth of patient-derived organoids (PDOs). (**A**,**B**) Using 3D CellTtiter-Glo assays, the impact of ERX-41 treatment on the viability of PDOs was evaluated (**A**). Representative pictures of PDOs cultured with or without ERX-41 treatment are shown (**B**). Data are represented as mean ± SEM. * *p* < 0.05; *** *p* < 0.001; **** *p* < 0.0001.

Recent research has indicated that patient-derived organoids (PDOs) serve as appropriate physiological ex vivo models for OCa, and PDOs demonstrate the preservation of genetic and phenotypic tumor heterogeneities [24]. We therefore examined the impact of ERX-41 on the growth of organoids derived from four primary OCa tissues. The results revealed that the treatment with ERX-41 significantly decreased their viability in a dose-dependent manner, compared to the treatment with the vehicle (Figure 5A,B). Overall, our findings indicate that ERX-41 hinders the viability of PDOs.

### 3.6. ERX-41 Reduced OCa Xenograft Tumor Growth In Vivo

**Figure 6:**
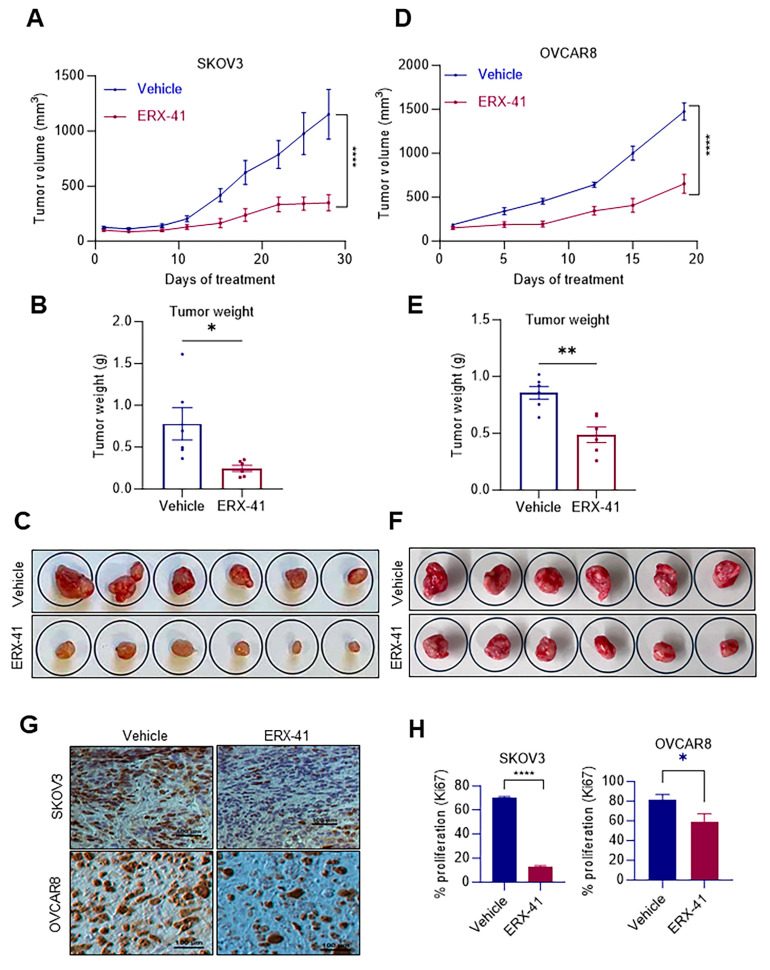
ERX-41 treatment suppresses OCa xenograft tumor growth in vivo. (**A**–**C**) SKOV3 cells were injected subcutaneously into SCID mice. Following tumor establishment, mice were randomly assigned to receive either vehicle (control) or ERX-41 (10 mg/kg body weight) five days a week through i.p. injection. Tumor volume was assessed at 3–5-day intervals (**A**). Tumor weights (**B**) and tumor pictures (**C**) are shown. (**D**–**F**) Following the establishment of subcutaneous OVCAR8 xenograft tumors, SCID mice were randomly assigned to receive either vehicle (control) or ERX-41 (10 mg/kg body weight) five days a week through i.p. injection. Tumor volume was assessed at 3–5-day intervals (**D**). Tumor weights (**E**) and tumor pictures (**F**) are shown. (**G**) SKOV3 and OVCAR8 xenograft tumor samples from the vehicle (control) and ERX-41 treatment groups were processed by IHC for Ki67 (proliferation marker) and quantitated (**H**). Scale bar represents 100 µm. Data are represented as mean ± SEM. * *p* < 0.05; ** *p* < 0.01; **** *p* < 0.0001.

To test the efficacy of ERX-41 on in vivo tumor progression, we used female SCID mice with established SKOV3 xenograft tumors. The mice were allocated at random to receive either a vehicle or ERX-41 once their tumor volume reached around 150 mm^3^. ERX-41 therapy showed significant reductions in tumor growth and tumor weights when compared to the group treated with the vehicle (Figure 6A–C). We further confirmed these findings by employing the OVCAR8 xenograft model. The results demonstrated that ERX-41 treatment effectively reduced OVCAR8 tumor volume and led to a reduction in tumor weights (Figure 6D–F). Further, the ERX-41-treated tumor exhibited a lower number of proliferating cells (Ki67 positive cells) compared to control tumors (Figure 6G,H). Overall, these findings indicate that ERX-41 exhibits strong antitumor effects on in vivo pre-clinical xenograft models of OCa.

## 4. Discussion

The standard of care chemotherapy is insufficient for treating OCa, as there is a high frequency of disease recurrence and low survival rates for patients with advanced OCa. There is thus an unmet need for new therapeutic approaches to target fundamental and critical pathways in OCa that will effectively cause OCa cell death and improve the survival of OCa patients. OCa cells display a heightened baseline level of ERS, and the expression of ER stress markers is strongly linked to OCa progression and prognosis. Several large-scale genetic screens have discovered components of the ER stress system to be vulnerable targets in OCa [10,11,12]. Therefore, identifying novel treatment targets that enhance the already activated system can diminish the defensive properties of the UPR, resulting in apoptosis and effectively overcoming the heterogeneity of OCa. Our findings indicate that ERX-41 is an effective agent for targeting ERS to kill OCa cells.

The cellular protein kinase R-like ER kinase (PERK) functions as one of the sensor of ERS and ameliorates enhanced ERS in OCa cells [25,26]. Critical to UPR is the activation of the eukaryotic translation initiation factor 2α (eIF2α), which decreases de novo protein translation and thus reduces stress on ER protein processing [13,27]. Several UPR-associated genes, the glucose-regulated protein (GRP78), PERK, and activating transcription factor 6 (ATF6) are induced at the transcriptional and translational levels [28] and restore homeostasis by compensating for enhanced ERS. High expression levels of GRP78 (also known as HSPA5/BiP), PERK, and ATF6 proteins are noted in OCa, compared to benign tissue, and the levels correlate with poor patient survival in high-grade serous-type OCa [29]. In this study, we found that ERX-41 induces ERS by activating UPR. Further, these results agree with our recent published study that showed that ERX-41 induced ERS in breast cancer cells [14].

Lysosomal acid lipase (LAL), encoded by the lipase A (*LIPA*) gene, hydrolyzes cholesteryl esters and triglycerides to produce free fatty acids and cholesterol in the cell [30]. Recent investigations have discovered a hitherto unknown role for *LIPA* in the endoplasmic reticulum, where it is involved in the process of protein folding [14]. Our previous study elucidated the precise molecular pathway through which ERX-41 triggers ER stress and the specific role of LIPA in mediating ERS. Specifically, ERX-41 inhibits the protein-folding activities mediated by LIPA in the ER, resulting in the accumulation of unfolded proteins. Consequently, this leads to ERS. These findings indicate that ERX-41 exacerbates the already activated ERS system in cancer cells, depleting its protective capabilities and inducing apoptosis [14]. A different study proposed that targeting LIPA could effectively decrease the stemness of TNBC [31]. The present study utilized tumor tissue microarray to demonstrate that OCa tumors have elevated levels of LIPA in comparison to normal ovarian tissues. The results of our TMA analysis align with the findings from TCGA data, indicating that there is an elevated expression of LIPA in OCa tissues.

This study utilized a varied collection of OCa cells, encompassing four distinct subtypes of OCa. We provided compelling evidence that ERX-41 diminishes cell viability, hampers the ability to form colonies, and triggers apoptosis. We further verified that the activity of ERX-41 on OCa cells is dependent on the presence of LIPA, as demonstrated by utilizing LIPA-KO OCa cells. The knockout of LIPA decreased the activity of ERX-41, whereas the re-expression of LIPA in KO cells restored the activity of ERX-41. Collectively, these findings indicate that ERX-41 exploits a common vulnerability in OCa cells by exhibiting a significant capacity to induce ERS in numerous OCa cells with distinct genetic backgrounds. Our study demonstrates the effectiveness of ERX-41 in decreasing the growth of OCa cells using PDOs and xenograft models.

## 5. Conclusions

This study examined the feasibility of developing a novel therapeutic approach that targets ERS, a prevalent vulnerability in OCa. Using tumor tissue array data, along with TCGA data, we demonstrated that LIPA is highly expressed in all four subtypes of OCa. By using fifteen distinct OCa models that encompass all sub-types of OCa, we successfully demonstrated that ERX-41 inhibits cell viability while promoting apoptosis. Mechanistic studies demonstrated that ERX-41 induces ERS and that LIPA mediates its effects. The efficacy of ERX-41 was validated in vivo and ex vivo through our xenograft and PDO experiments, respectively. Our findings suggest that using ERX-41 to target LIPA could serve as a promising and efficient treatment for all four subtypes of OCa cells, potentially addressing tumor heterogeneity.

## Data Availability

All data generated for this study are included within this article.

## Supplementary Materials

The following supporting information can be downloaded at: https://www.mdpi.com/article/10.3390/cancers16030500/s1, Figure S1: The efficacy of ERX-41 correlates with the expression of LIPA. (A) Western blot analysis shows the expression of LIPA in multiple ovarian cancer cells. (B) Bar graphs show the densitometric quantification of Western blots. (C) Scatter plot shows the simple linear regression analysis between the LIPA expression and the percent growth inhibition of OCa cells at 125 nM of ERX41. (D,E) Unprocessed scans of Western blots. Cropped sections used as figures in the manuscript are marked as a box; Figure S2: (A) Unprocessed scans of agarose gel: ES2, OVCAR3, OCa39; (B–D) Unprocessed scans of Western blots: (B) OCa39, OVCAR8; (C) A2780, SKOV3; (D) OV90; Bar graphs show the densitometric quantification of Western blots. Figure S3: (A) Unprocessed scans of Western blots: SKOV3; (B) Unprocessed scans of agarose gel: SKOV3-WT, SKOV3-LIPA-KO; Bar graphs show the densitometric quantification of Western blots; Table S1: List of primary OCa cells used for the study; Table S2: List of RT-qPCR primers used for the study.
