# Supplementary material for: Novel LIPA-Targeted Therapy for Treating Ovarian Cancer
Source: Cancers (Basel). 2024 Jan 24;16(3):500. doi: 10.3390/cancers16030500 (PMC10854701; doi:10.3390/cancers16030500)
Supplement: Supplementary file 1 [file cancers-16-00500-s001.zip › cancers-2825652-supplementary.pdf]

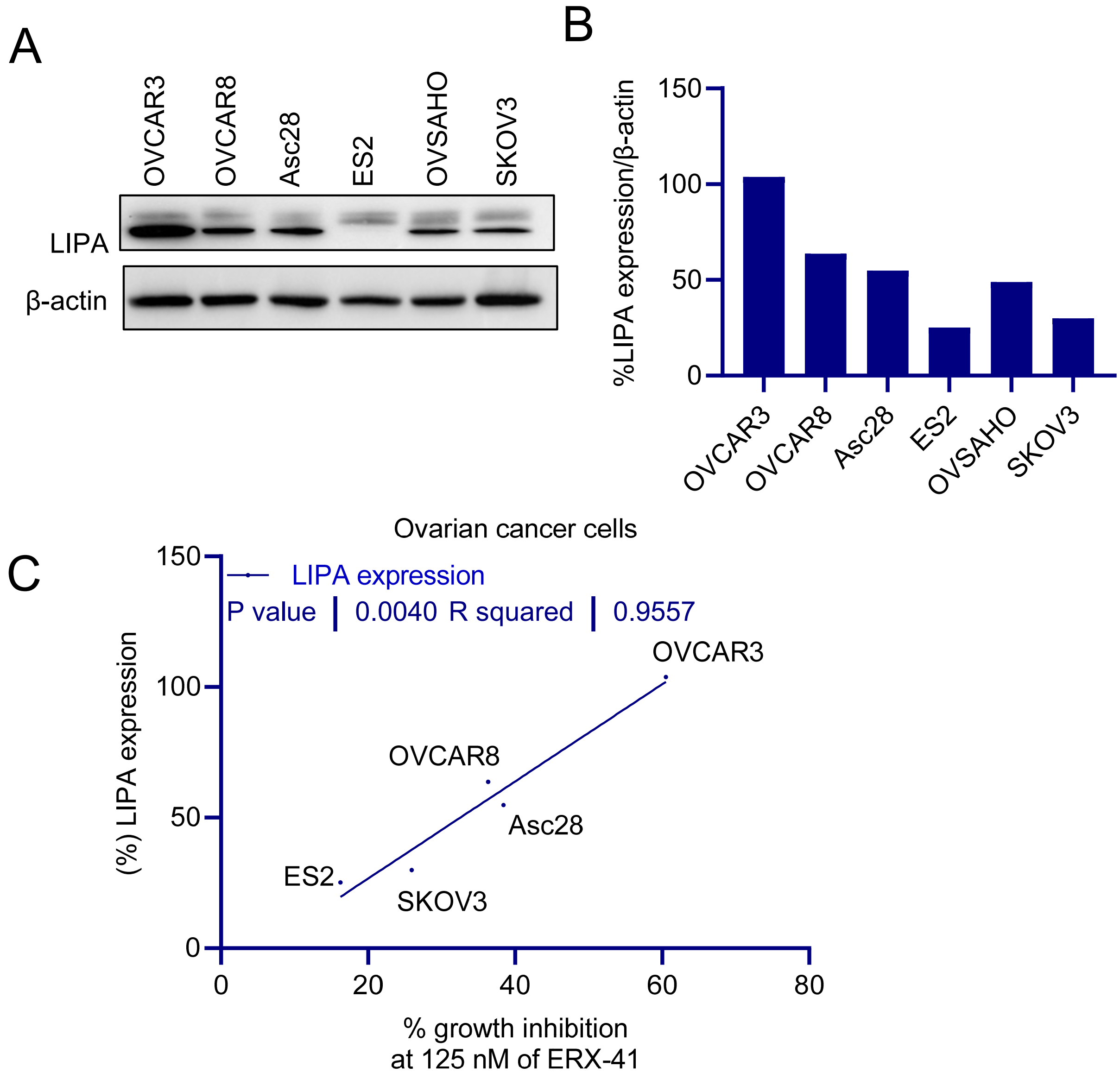

Supplementary Figure S1. The efficacy of ERX-41 correlates with the expression of LIPA. (A) Western blot analysis of multiple ovarian cancer cells shows the expression of LIPA in multiple ovarian cancer cells. (B) Bar graphs show the densitometric quantification of Western blots. (C) Scatter plot shows the simple linear regression analysis between the LIPA expression and the percent growth inhibition of OCa cells at 125 nM of ERX41.

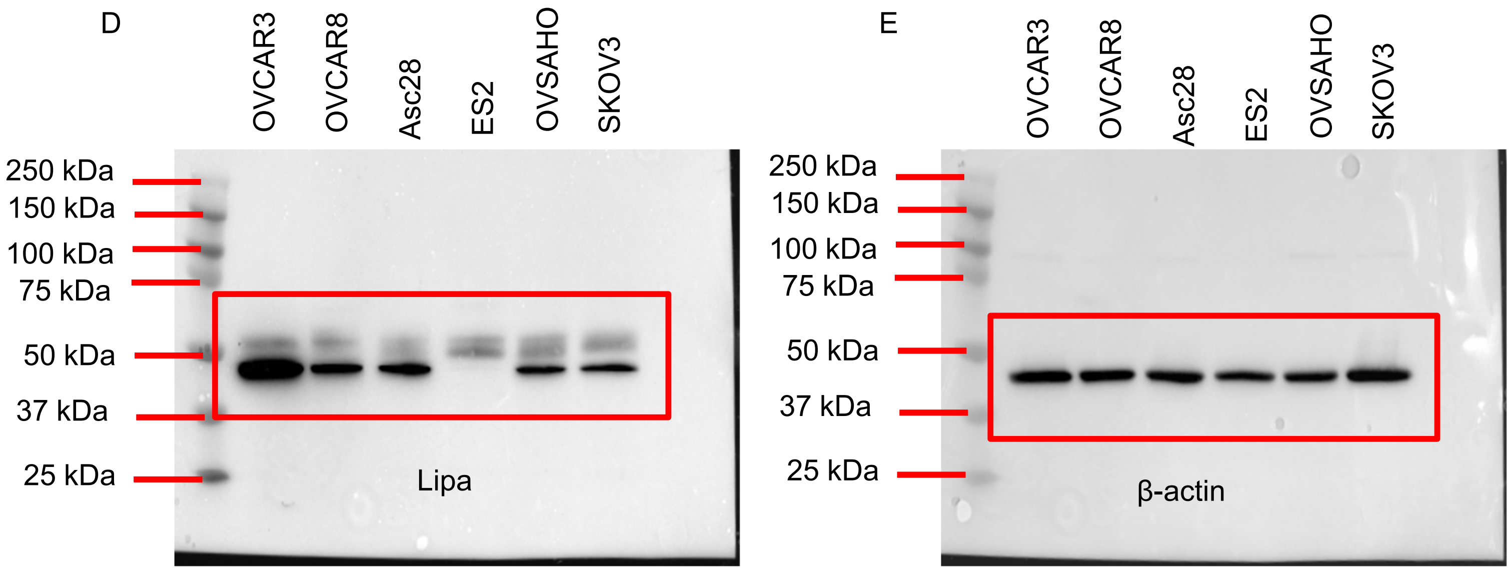

Supplementary Figure S1. (D,E) Unprocessed scans of Western blots. Cropped sections used as figures in the manuscript are marked as a box.

Supplementary Figure S2A

ES2

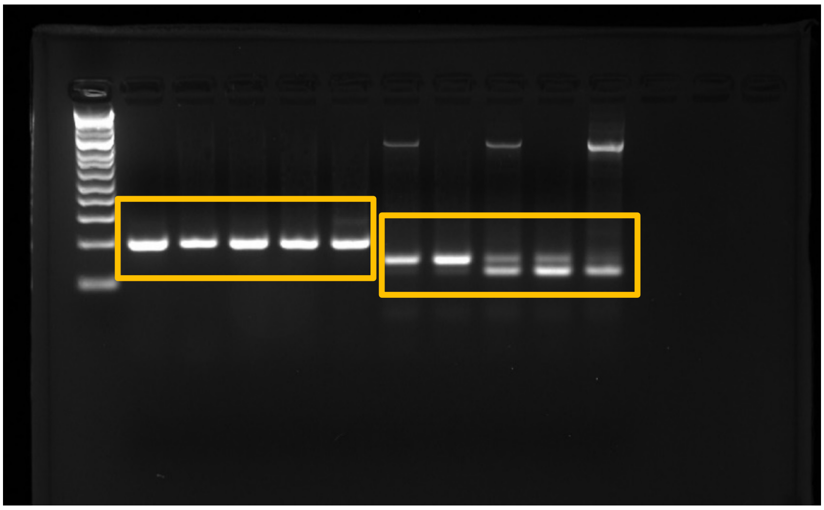

OVCAR3

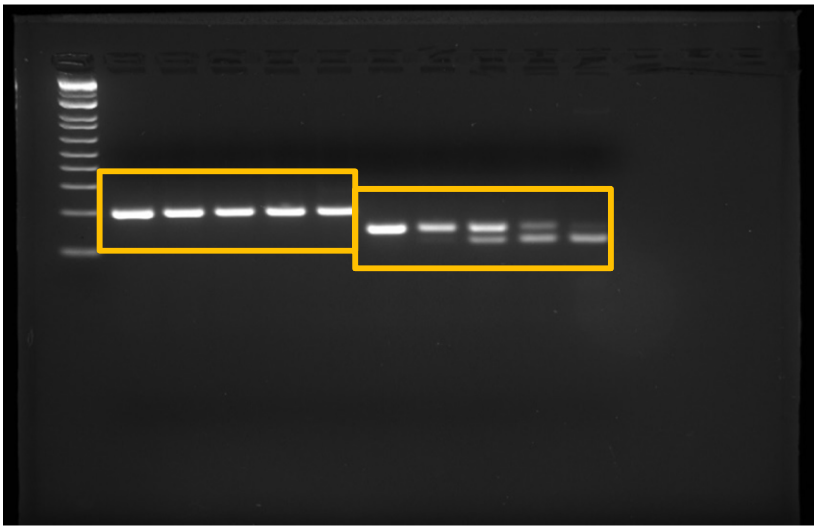

OCa39

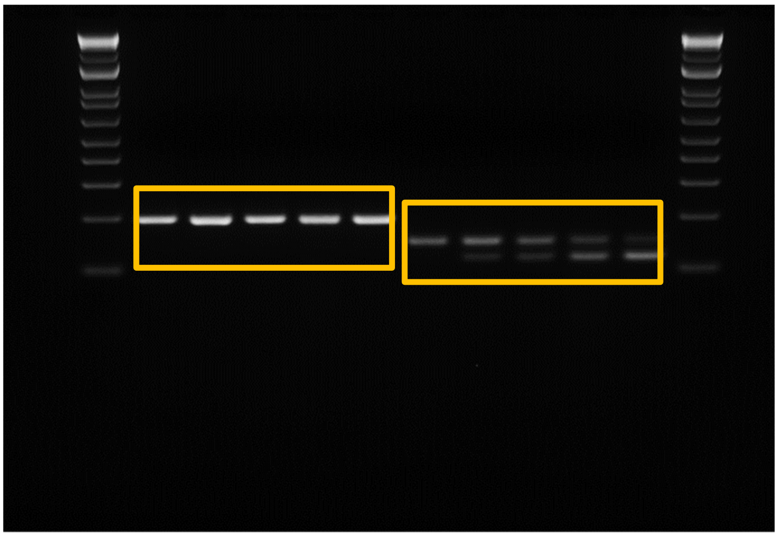

Supplementary Figure S2. (A) Unprocessed scans of agarose gel. Cropped sections used as figures in the manuscript are marked as a box.

Supplementary Figure S2B

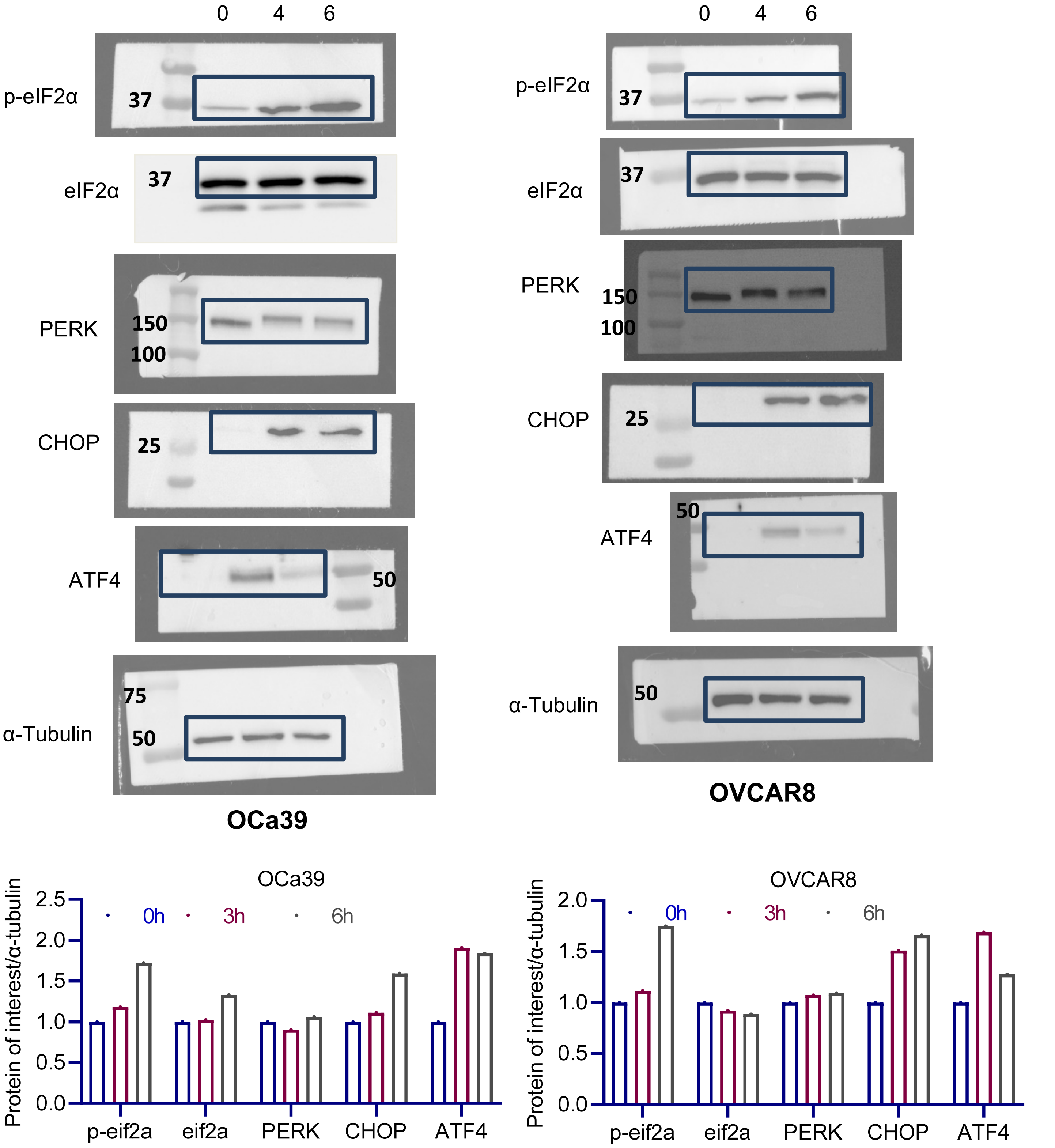

Supplementary Figure S2. (B) Unprocessed scans of Western blots were shown in upper panel. Cropped sections used as figures in the manuscript are marked as a box. Densitometric quantification of western blots were shown as bar graphs in the lower panel.

Supplementary Figure S2C

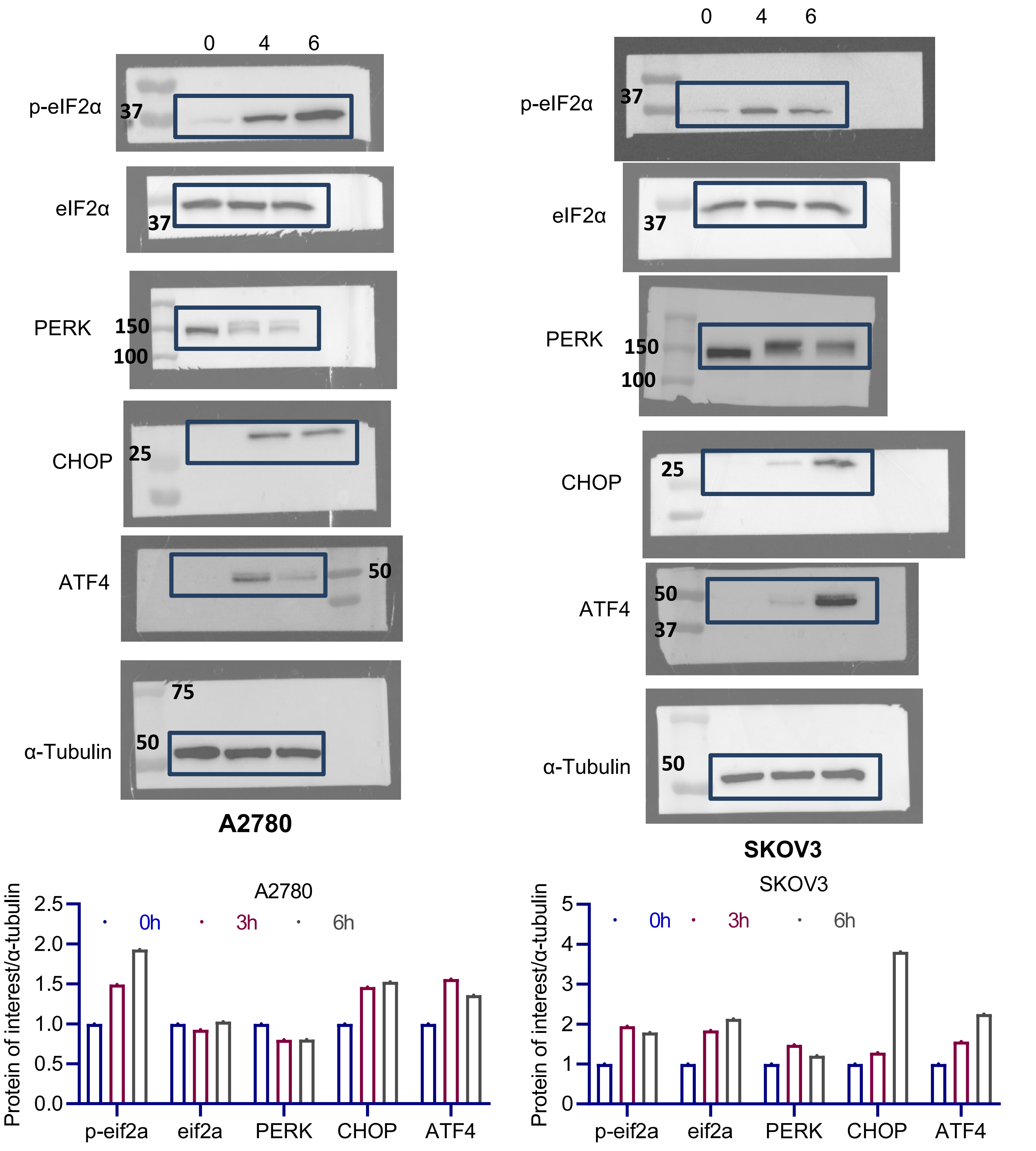

Supplementary Figure S2. (C) Unprocessed scans of Western blots were shown in upper panel. Cropped sections used as figures in the manuscript are marked as a box. Densitometric quantification of western blots were shown as bar graphs in the lower panel.

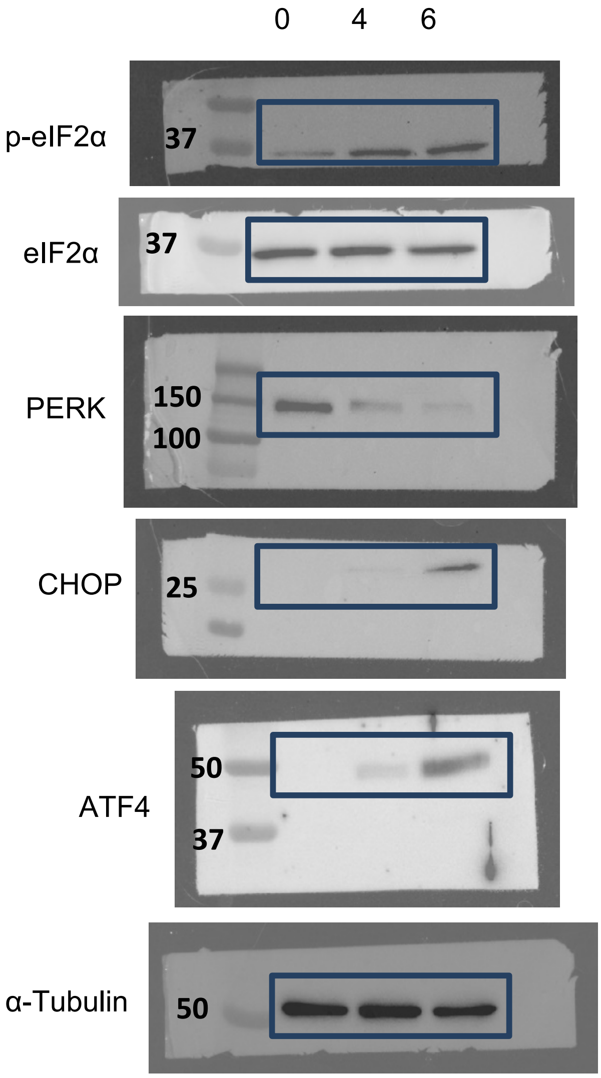

OV90

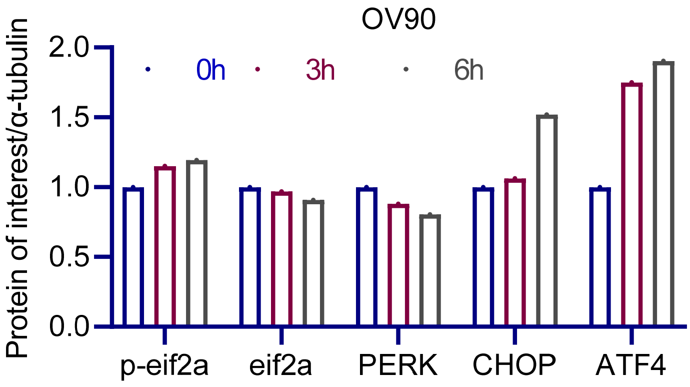

Supplementary Figure S2. (D) Unprocessed scans of Western blots were shown in upper panel. Cropped sections used as figures in the manuscript are marked as a box. Densitometric quantification of western blots were shown as bar graphs in the lower panel.

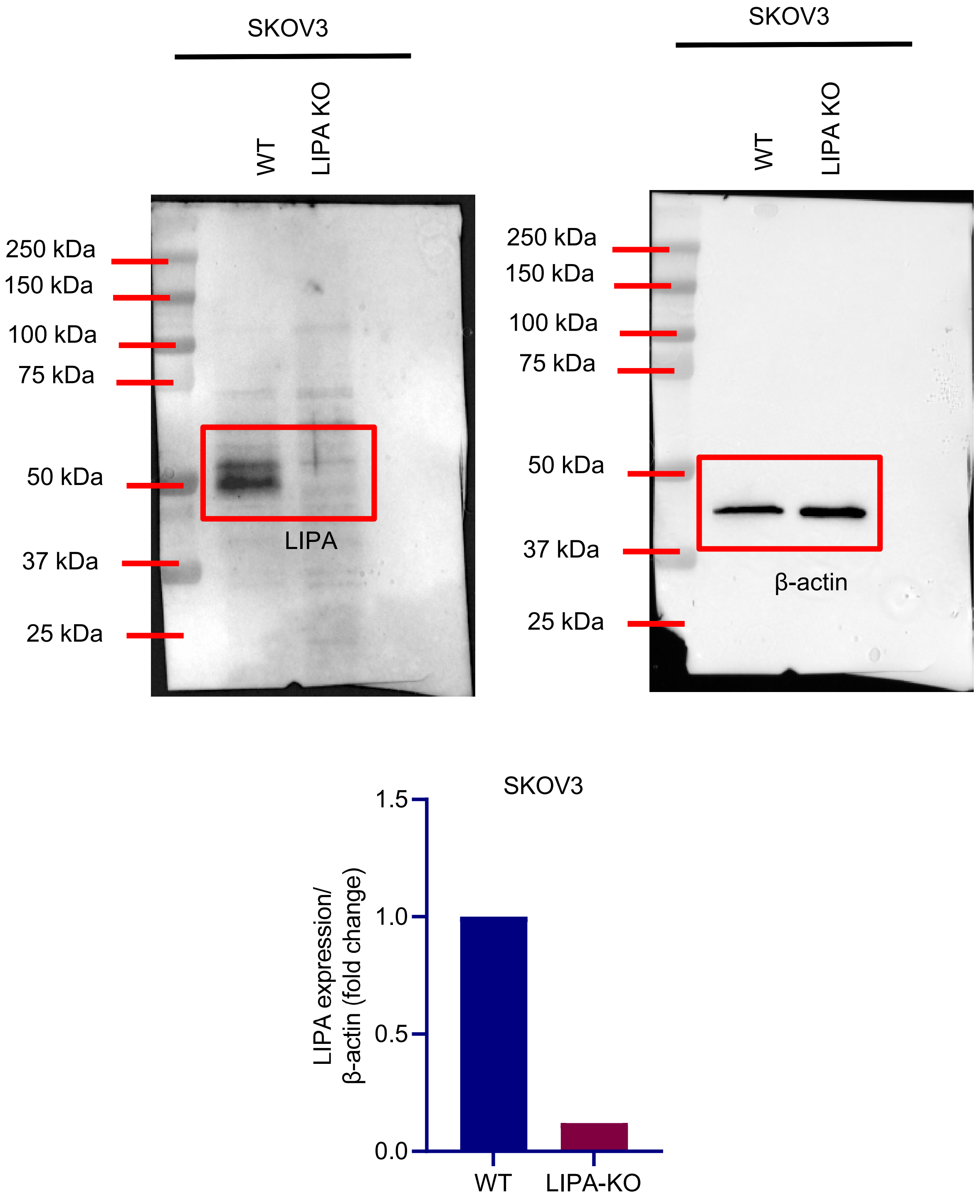

Supplementary Figure S3. (A) Unprocessed scans of Western blots were shown in upper panel. Cropped sections used as figures in the manuscript are marked as a box. Densitometric quantification of western blots were shown as bar graphs in the lower panel.

Supplementary Figure S3B

SKOV3-WT

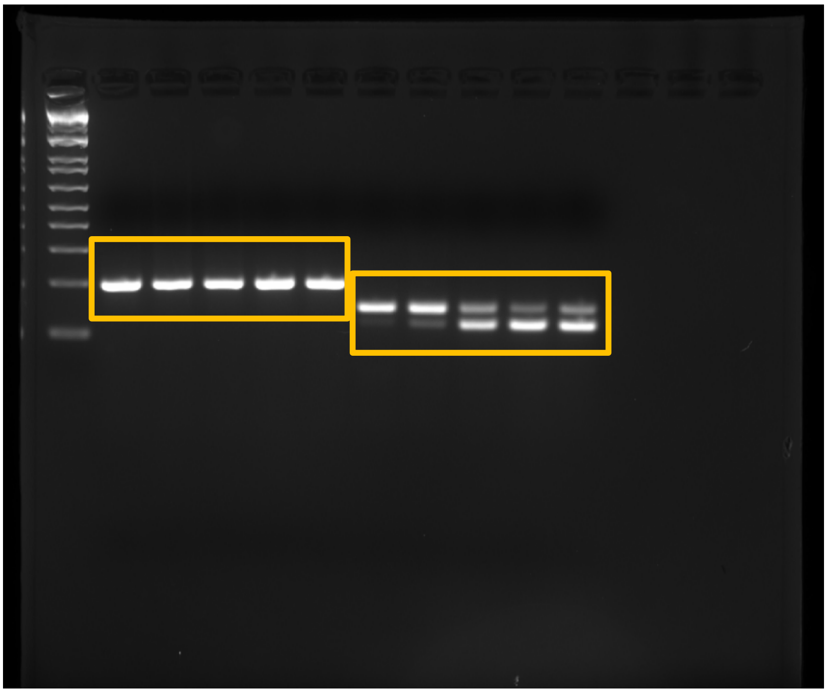

SKOV3-LIPA-KO

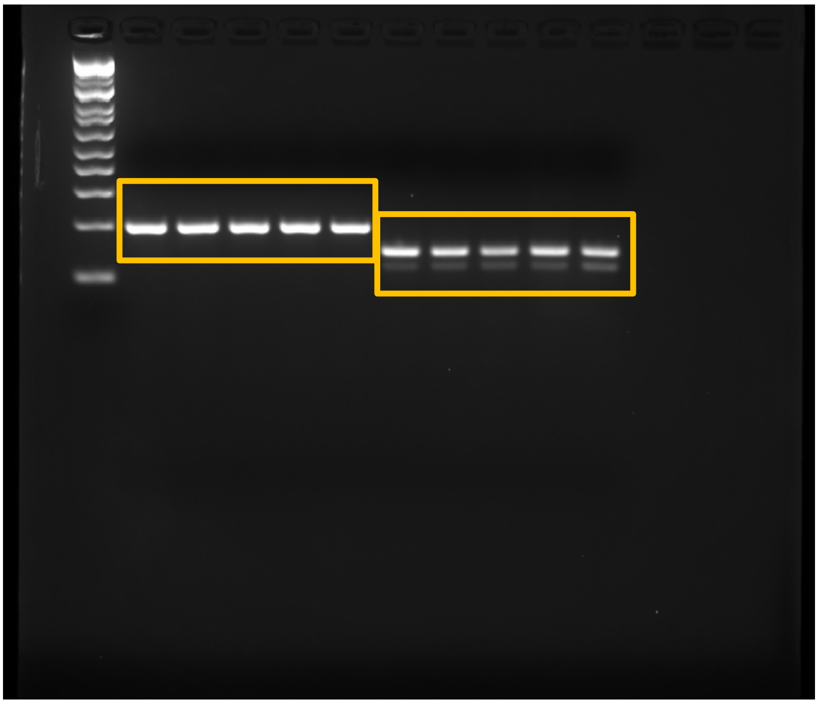

Supplementary Figure S3. (B) Unprocessed scans of agarose gel. Cropped sections used as figures in the manuscript are marked as a box.

Supplementary Table S1. List of primary OCa cells used for the study.

Number	Histology	Age	Grade	Stage	Stains
Asc25	HGSOC	48	High	IIIC	Positive for WT1, PAX8, ER and P16, Negative for p53 and Napsin
Asc28	HGSOC	78	High	IV	Positive for p53, WT1, ER and PAX8, Calretinin highlights mesothelial cells
OCa30	HGSOC	48	High	IIIC	Not available
OCa/Asc39	HGSOC	62	High	IB	Positive for WT1 and p53, CA125 (luminal staining)
OCa49	Clear cell	54	Grade 3	IIIC	PAS- small amount of glycogen, Positive for ER, CD10(focal +), PAX8 (patchy) Negative for PR and Napsin
Asc34	HGSOC	54	High	IIIC	Positive for WT1, PAX8, ER and P16, Negative for p53
OCa38	HGSOC	52	High	IVB	Not available
OCa55	HGSOC	56	High	IIIC	WT1 positive, ER positive, patchy staining for p16, p53 null
OCa58	LGSOC	53	Low	IIIC	WT1 diffusely positive, p53 WT, Ki67<10%
OCa60	Endometrioid	54	Grade 2	IA	ER/PR positive

Supplementary Table S2. List of RT-qPCR primers used for the study.

Gene name	Forward Primer	Reverse Primer
XBP1	CCCTCCAGAACATCTCCCCAT	ACATGACTGGGTCCAAGTTGT
ATF3	CCTCTGCGCTGGAATCAGTC	TTCTTTCTCGTCGCCTCTTTTT
DDIT3	GGAAACAGAGTGGTCATTCCC	CTGCTTGAGCCGTTCATTCTC
ATF4	CTCCGGGACAGATTGGATGTT	GGCTGCTTATTAGTCTCCTGGAC
HSPA5	CATCACGCCGTCCTATGTCG	CGTCAAAGACCGTGTTCTCG
GAPDH	GGAGCGAGATCCCTCCAAAAT	GGCTGTTGTCATACTTCTCATGG
